# An Uncommon Case of Lichen Spinulosus in an Adult Patient Clinically Mmimicking Folliculotropic Mycosis Fungoides

**DOI:** 10.7759/cureus.8572

**Published:** 2020-06-12

**Authors:** Maryam Aghighi, Tatsiana Pukhalskaya, Sylvana Brickley, Bruce Smoller

**Affiliations:** 1 Pathology, Saint Barnabas Medical Center, Robert Wood Johnson Barnabas Health, Livingston, USA; 2 Pathology, University of Rochester Medical Center, Rochester, USA; 3 Dermatology, University of Rochester Medical Center, Rochester, USA; 4 Pathology and Dermatology, University of Rochester School of Medicine and Dentistry, Rochester, USA

**Keywords:** lichen spinulosus, perifollicular lymphohistiocytic infiltrate

## Abstract

Lichen spinulosus (LS) is an uncommon skin condition mostly in children and adolescents but uncommon in adults. It presents as a group of hypopigmented or skin-colored follicular papules and keratotic spines with a sandpaper-like appearance. There is a lymphohistiocytic infiltrate in the dermis centered around hair follicles. We present a rare case of LS in a 52-year-old woman with a rough, bumpy, itchy rash affecting the trunk and extremities. Her rash consisted of clusters of hyperkeratotic follicular-based spiny papules. Histologic sections demonstrated several dilated hair follicles filled with keratotic plugs surrounded by a dense perifollicular lymphohistiocytic infiltrate, particularly at the level of the infundibula, that extended into the follicular epithelium.

## Introduction

Lichen spinulosus (LS) is a rare dermatosis with an unknown etiology. It is most common in children and adolescents but occurs very rarely in adults [1]. It may have a genetic predisposition or be associated with gold, arsphenamine, thallium, diphtheria toxin, atopy, lithium therapy, Crohn's disease, Hodgkin disease, human immunodeficiency virus (HIV) or alcoholism [2-5]. Additionally, vitamin A deficiency is associated with LS [1]. It presents as a group of 2-5 cm hypopigmented or skin-colored follicular papules and keratotic spines with a sandpaper-like appearance [6]. The lesions usually erupt on various regions of the skin and remain for weeks to months. The histologic findings consist of a lymphohistiocytic infiltrate in the dermis centered around hair follicles. We report an uncommon case of LS occurring in an adult patient in whom the main clinical concern was for folliculotropic mycosis fungoides.

## Case presentation

**Figure 1:**
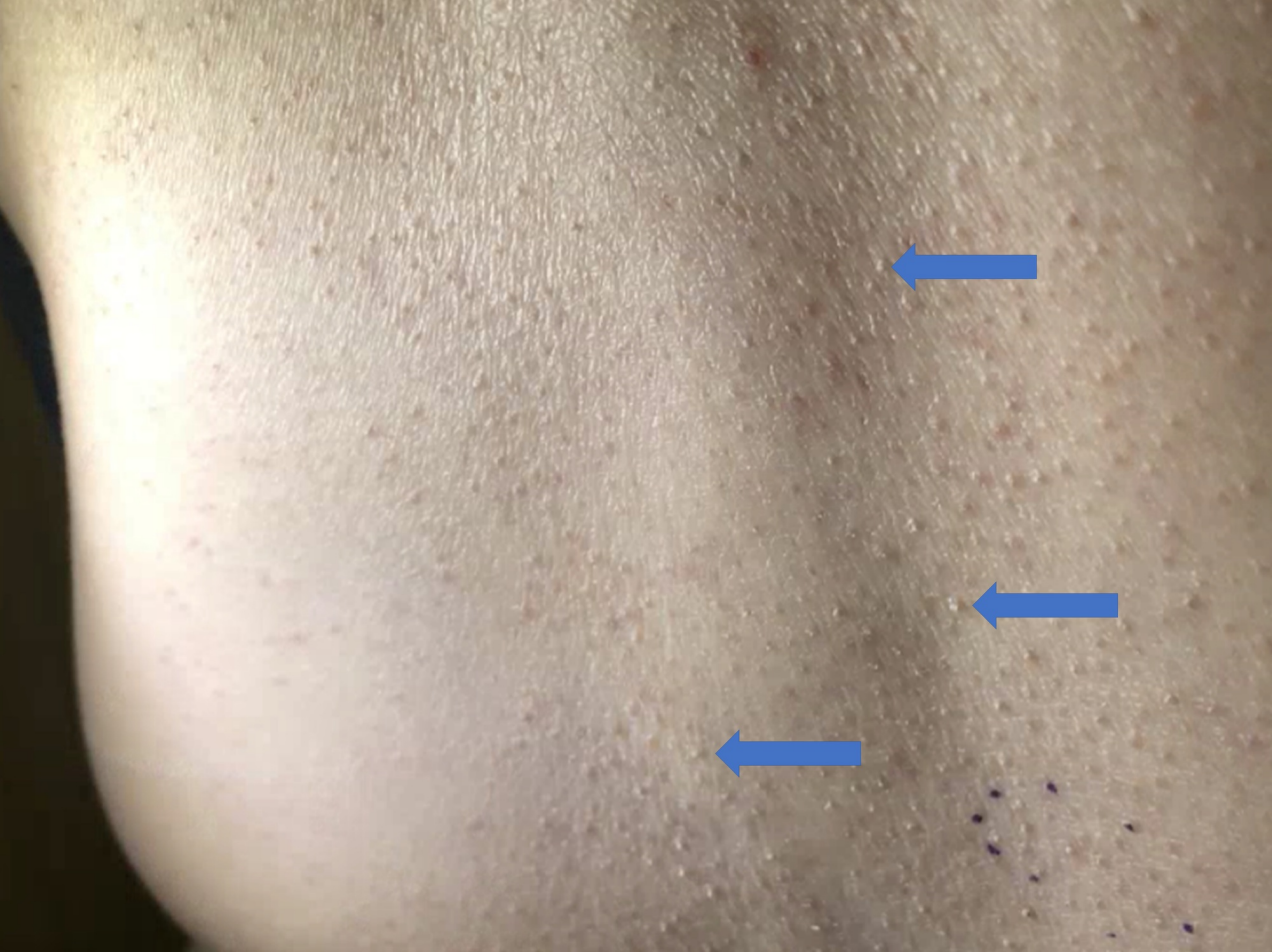
Clinical presentation of lichen spinulosus (LS): clusters of hyperkeratotic follicular based spiny papules on the lower back of the patient (blue arrows)

A 52-year-old female presented to the dermatology clinic with a rough, bumpy minimally itchy rash affecting the lower back, bilateral arms, abdomen, anterior thighs, and shins. She had a history of hypertension, hypothyroidism, vitamin D deficiency, vitreous hemorrhage, bilateral carotid artery dissection, dissecting hemorrhage of the left vertebral artery, cardiomyopathy, and subarachnoid hemorrhage. The cutaneous eruption had been present for about a year. She had not used any topical steroids or any other treatment before her dermatologic visit. She had no personal or family history of skin cancer. She reported no smoking history or alcohol consumption. She did not have any fevers, chills, weight loss, nausea, vomiting, diarrhea, or mouth sores. Her eruption consisted of clusters of hyperkeratotic follicular based spiny papules and scattered in a widespread distribution on the anterior bilateral thighs, lower back, abdomen, and bilateral forearms as demonstrated in Figure 1.

Vitamin A supplementation (10,000 units daily for two months) for slight hypovitaminosis A (level 30 mgc/dL, reference range 38-98 mcg/dL) led to no lesion improvement. Twice daily Urea 20% cream was added. A shave biopsy was performed with clinical concern for folliculotropic mycosis fungoides, scurvy and LS.

**Figure 2:**
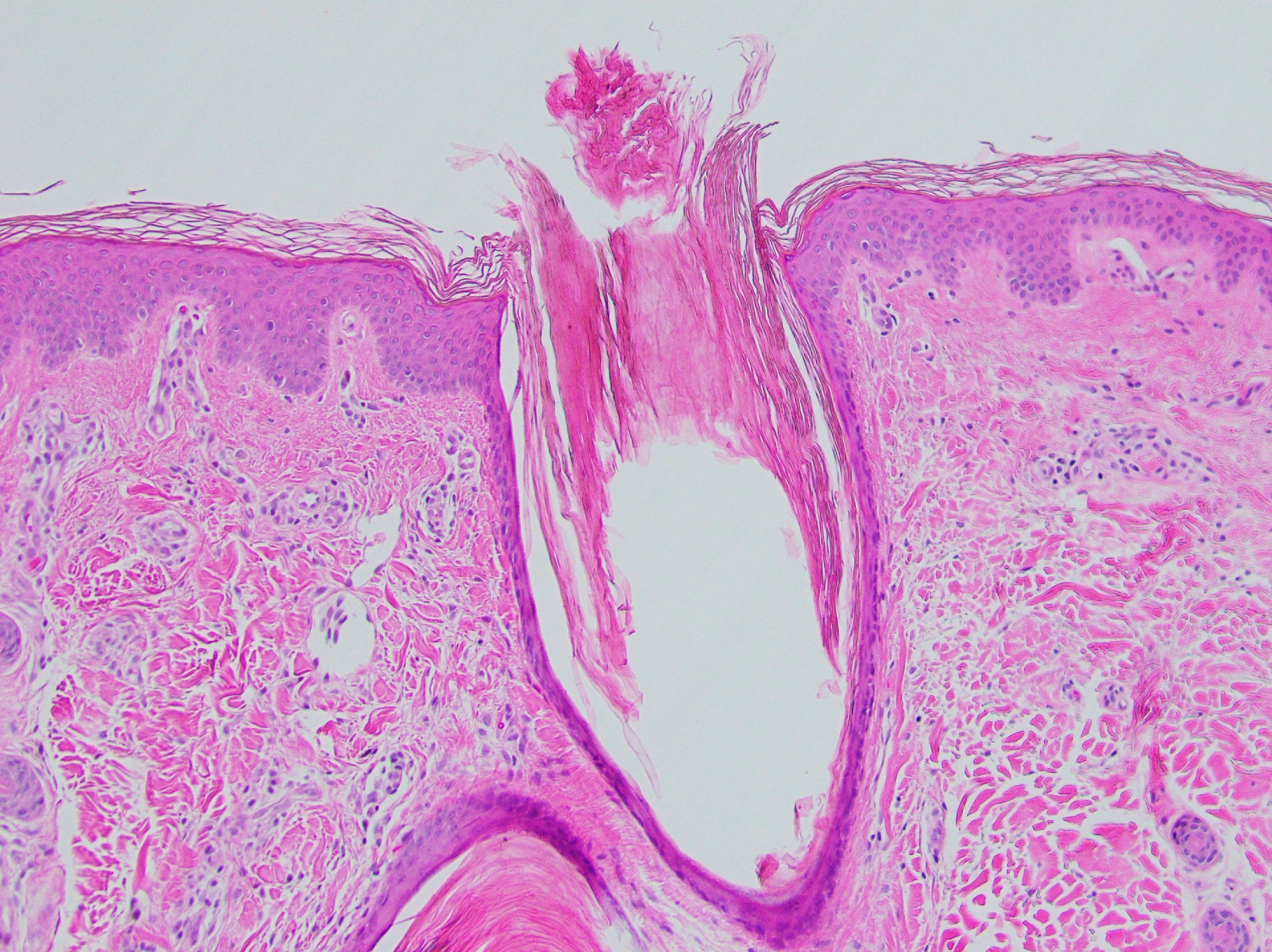
Hematoxylin and eosin (H&E) staining (100x) identifying dilated hair follicle filled with keratotic plugs surrounded by dense perifollicular lymphohistiocytic inflammatory infiltrates

**Figure 3:**
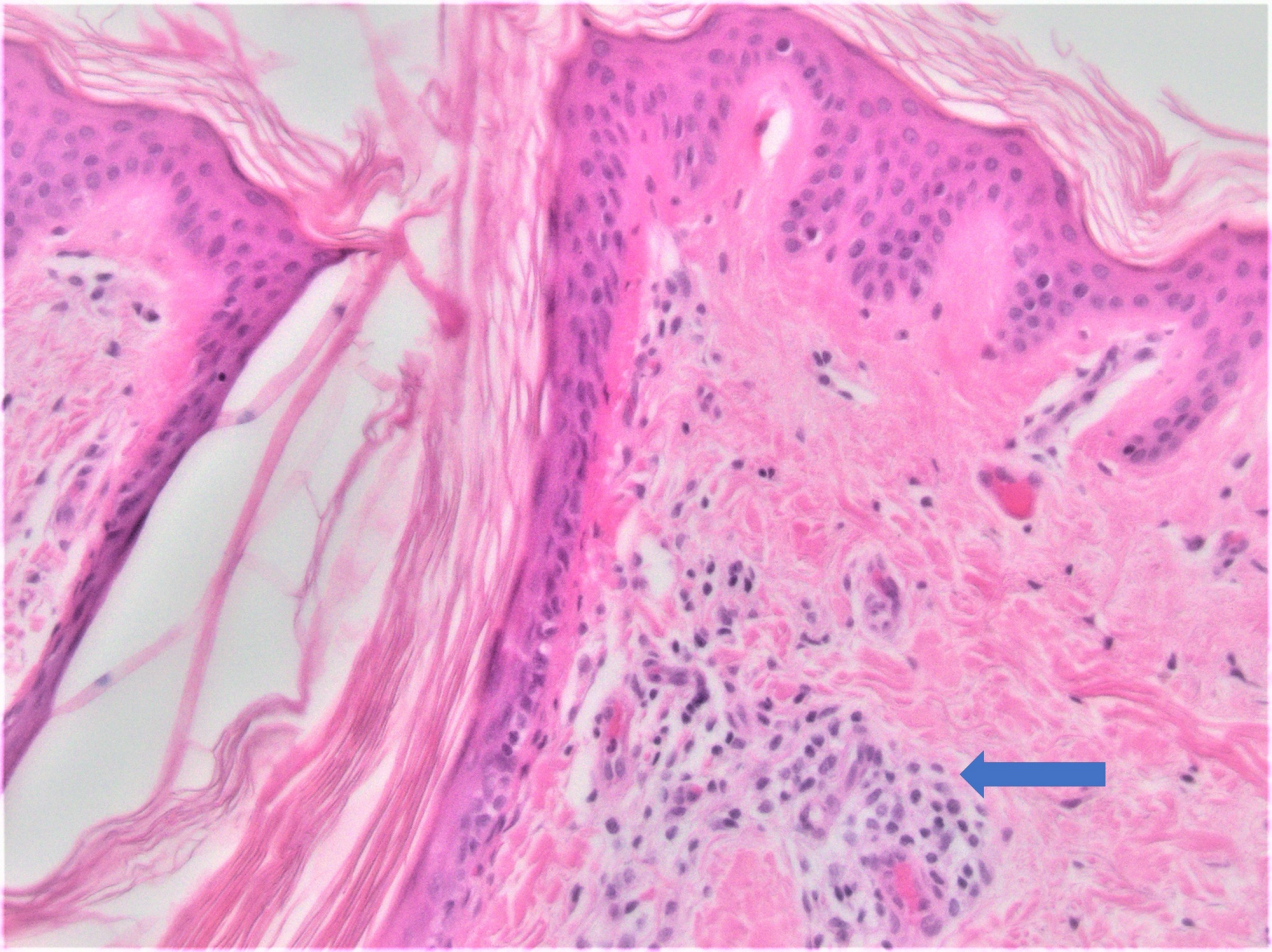
Hematoxylin and eosin (H&E) staining (200x) identifying lymphohistiocytic inflammatory infiltrates around the hair follicle (blue arrow)

**Figure 4:**
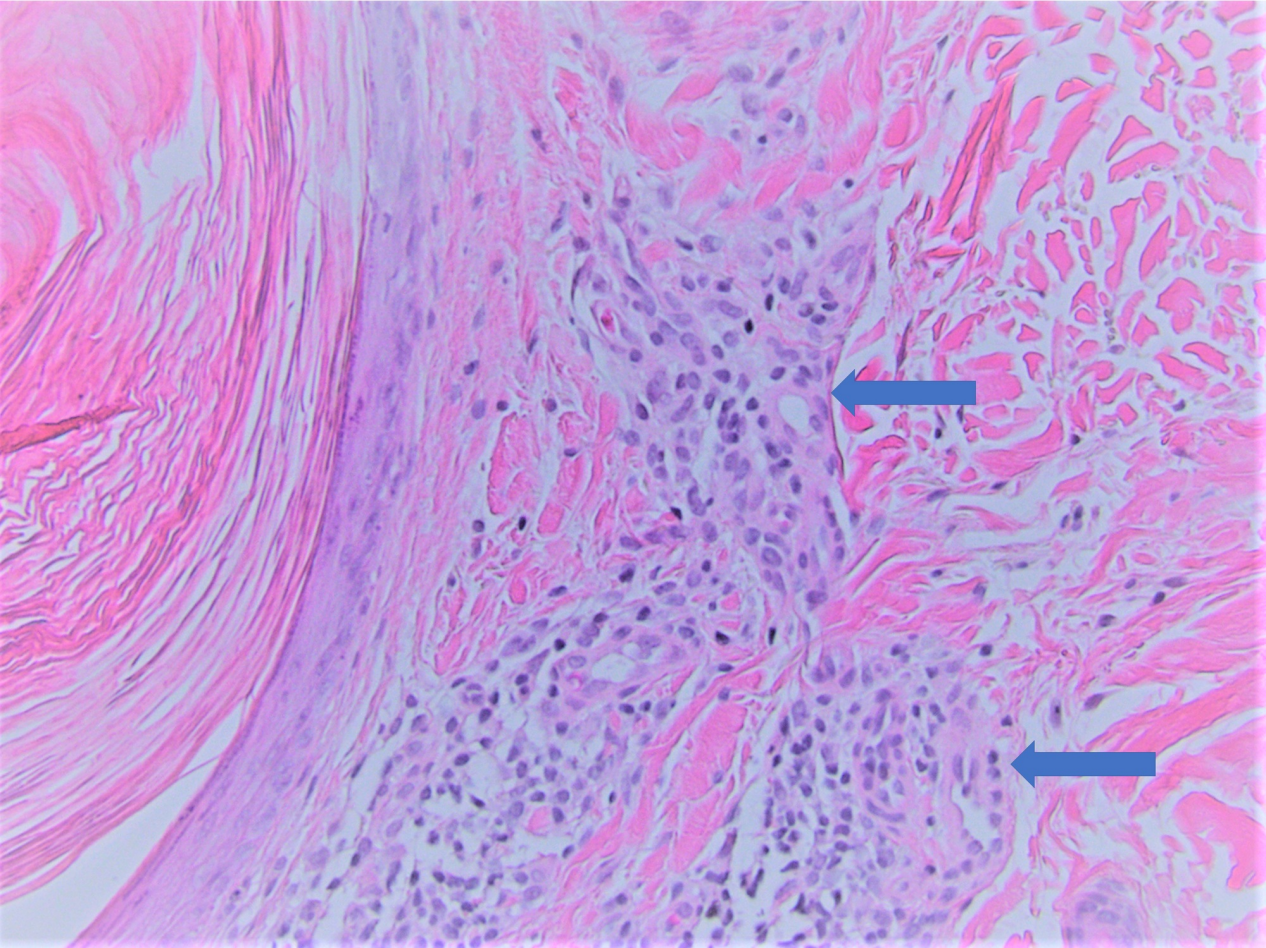
Hematoxylin and eosin (H&E) staining (200x) identifying lymphohistiocytic inflammatory infiltrates around the hair follicle (blue arrows)

Histologic sections demonstrated several dilated hair follicles filled with keratotic plugs and surrounded by dense perifollicular lymphohistiocytic inflammatory infiltrates, particularly at the level of the infundibula. The lymphocytic infiltrate extended into the follicular epithelium with concomitant spongiosis. There was mild perifollicular fibrosis and noticeable atrophy of the sebaceous glands as illustrated in Figures 2-4. No atypical lymphocytes or epidermotropism were identified. These findings are those classically described in LS. Given the clinical concerns, a T-cell gene rearrangement study was performed and failed to demonstrate T-cell clonality.

## Discussion

LS is a rare dermatosis similar to keratosis pilaris more commonly observed in children and young adults and occurs mostly in male patients. It is rarely observed in adults and elderly patients. Previously, a few cases of LS have been reported in the literature as summarized in Table 1.

**Table 1:** Previously reported LS cases LS: Lichen Spinulosus, M: Male, F: Female

Authors	Publication year	Number of cases and gender	Age	Locations
Al Hawsawi et al. [6]	2015	1 M	12	Lower back
Uehara et al. [7]	2015	1 M	69	Trunk and limbs
Litao et al. [8]	2014	1 F	7	Trunk, extremities, hands and face
Sobjanek et al. [9]	2014	1 M	8	Knees and shins
Venkatesh et al. [10]	2012	1 M	4	Forehead, neck, abdomen, back, hips, groin and extensor extremities
Seo et al. [11]	2009	1 F	50	
Kabashima et al. [3]	2008	1 M	59	Forehead
Kim et al. [12]	2008	1 M	7	Submental area
Oh et al. [13]	2005	1 M	7	Both elbows and knees
Kim et al. [14]	2001	1 M	8	
Mittal et al. [15]	1997	1 F	8	Neck, trunk, buttocks and extensors of limbs
Kano et al. [16]	1995	1 F	61	Back, intertriginous areas of groin, inframammary and left axilla
Cohen et al. [2]	1991	1 M	31	Face and trunk
Friedman [1]	1990	14 M, 21 F	17.8 $\pm$ 9.5	Arms and legs, back, chest, face and neck
Tuyp et al. [17]	1984	1 M	15	Knees, elbows and lower legs

LS shares some clinical and histologic similarity to folliculotropic mycosis fungoides, which may also present with an acne-like eruption and a peri-follicular lymphocytic infiltrate [18]. As in our case wherein, the eruption was longstanding, clinical, histological, and molecular observations may be required to discriminate LS from this variant of mycosis fungoides. Lymphocyte atypia, follicular epidermotropism, and evidence for T-cell receptor gene rearrangements may be used to make this distinction. None of these findings were observed in the current case.

Topical keratolytics and emollients including salicylic acid, vitamin A, tretinoin, tacalcitol, adapalene, and urea are commonly used to treat LS [4,7-8,19-20]. If left untreated, the lesions may resolve on their own after a few weeks to months [10]. For patients with an underlying problem, treatment of the disease may improve LS [3]. Additionally, lesion recurrence has been reported [4].

## Conclusions

LS is a less common skin dermatosis which rarely arises in adult patients. Clinical examination, histological evaluation, and molecular studies may be required to differentiate LS from other entities such as folliculotropic mycosis fungoides.

## References

[1] Friedman SJ (1990). Lichen spinulosus: clinicopathologic review of thirty-five cases. J Am Acad Dermatol.

[2] Cohen SJ, Dicken CH (1991). Generalized lichen spinulosus in an HIV-positive man. J Am Acad Dermatol.

[3] Kabashima R, Sugita K, Kabashima K, Nakamura M, Tokura Y (2009). Lichen spinulosus in an alcoholic patient. Acta Derm Venereol.

[4] Tilly JJ, Drolet BA, Esterly NB (2004). Lichenoid eruptions in children. J Am Acad Dermatol.

[5] Boyd AS (1989). Lichen spinulosus: case report and overview. Cutis.

[6] Al Hawsawi K, Almehmadi K, Alraddadi B, Aljuhani O (2015). Lichen spinulosus: case report and review of literatures. J Health Sci.

[7] Uehara A, Abe M, Shimizu A, Motegi SI, Amano H, Ishikawa O (2015). Successful treatment of lichen spinulosus with topical adapalene. Eur J Dermatol.

[8] Litao Litao, M. K. S.; Amer A. (2020). Lichen spinulosus in a young girl. patientcareonline.

[9] Sobjanek M, Sikorska M, Sokolowska-Wojdylo M, Nowicki R (2014). Lichen spinulosus. Przegl Dermato.

[10] Venkatesh A, Dupuis E, Prajapati V, Rao J (2012). Generalized lichen spinulosus in a 4-year-old boy without systemic disease. Arch Dermatol.

[11] Seo JW, Sim HS, Choi JH, Lee SK (2009). A case of lichen spinulosus arising at the site of antecedent seborrheic dermatitis. Korean J Dermatol.

[12] Kim CY, Park SY, Oh CW (2008). A case of lichen spinulosus. Korean J Dermatol.

[13] Oh DH, Park KT, Kim JS, Yu HJ (2005). A case of lichen spinulosus with an histologic finding of follicular mucinosis. Ann Dermatol.

[14] Kim GN, Yu DS, Son SW, Kim AR, Kim IW (2001). A case of lichen spinulosus. Korean J Dermatol.

[15] Mittal RR, Kaur M (1997). Lichen spinulosus. Indian J Dermatol.

[16] Kano Y, Orihara M, Yagita A, Shiohara T (1995). Lichen spinulosus in a patient with Crohn's discase. Int J Dermatol.

[17] Tuyp E, McLeod W, Boyko W (1984). Lichen spinulosus with immunofluorescent studies. Cutis.

[18] van Santen S, Jansen PM, Quint KD, Vermeer MH, Willemze R (2020). Plaque stage folliculotropic mycosis fungoides: histopathologic features and prognostic factors in a series of 40 patients. J Cutan Pathol.

[19] Forman SB, Mac Hudgins E, Blaylock WK (2007). Lichen spinulosus: excellent response to tretinoin gel and hydroactive adhesive applications. Arch Dermatol.

[20] Kim SH, Kang JH, Seo JK, Hwang SW, Sung HS, Lee D (2010). Successful treatment of lichen spinulosus with topical tacalcitol cream. Pediatr Dermatol.

